# Mechanism of pentoxifylline mediated down-regulation of killer lineage cell functions

**DOI:** 10.1155/S0962935193000535

**Published:** 1993

**Authors:** R. S. Parhar, P. Ernst, K. Sheth, M. Einspenner, S. Al-Sedairy

**Affiliations:** 1 Department of Biological and Medical Research King Faisal Specialist Hospital and Research Centre P.O. Box 3354 Riyadh 11211 Saudi Arabia; 2 Department of Oncology King Faisal Specialist Hospital and Research Centre P.O. Box 3354 Riyadh 11211 Saudi Arabia; 3 Department of Pathology King Faisal Specialist Hospital and Research Centre P.O. Box 3354 Riyadh 11211 Saudi Arabia

## Abstract

The authors reported recently that endotoxaemia mediated elevated levels of tumour necrosis factor (TNF-α) and interleukin-1α (IL-1α) were involved in the pathophysiology of acute heat stroke patients. Pentoxifylline (PTX) is known to modulate neutrophil functions. In the present study the effects of PTX on lipopolysaccharide (LPS) and cytokine induced T-cell and macrophage (ΦM) activation, and on natural killer (NK) cell and lymphokine activated killer (LAK) cell mediated cytotoxicity were examined. Finally, the effect of PTX on the expression of adhesion molecules (LFA-1, Mac-1 and ICAM-1), and cytokine (IL-1α, IL-2, TNF-α, IL-6 and IFN-γ) production and their surface receptor expression in response to LPS activation was investigated. PTX free cultures served as a control. Results revealed that PTX can down-regulate all the above-mentioned immunological parameters in a dosedependent manner. These findings might have far reaching clinical implications.


Mediators of Inflammation 2, 379–384 (1993)

### Mechanism of pentoxifylline mediated down-regulation of killer lineage cell functions*

R. S. Parhar,[1CA] P. Ernst,[2] K. Sheth,[3] M. Einspenner[1] and S. Al-Sedairy[1]

[1]Department of Biological and Medical Research, [2]Department of Oncology, [3]Department of Pathology, King Faisal Specialist Hospital and Research Centre, P.O. Box 3354, Riyadh 11211, Saudi Arabia

[CA] Corresponding Author

**THE authors reported recently that endotoxaemia mediated elevated levels of tumour necrosis factor (TNF-α) and interleukin-1α (IL-1α) were involved in the pathophysiology of acute heat stroke patients. Pentoxifylline (PTX) is known to modulate neutrophil functions. In the present study the effects of PTX on lipopolysaccharide (LPS) and cytokine induced T-cell and macrophage (φM) activation, and on natural killer (NK) cell and lymphokine activated killer (LAK) cell mediated cytotoxicity were examined. Finally, the effect of PTX on the expression of adhesion molecules (LFA-1, Mac-1 and ICAM-1), and cytokine (IL-1α, IL-2, TNF-α, IL-6 and IFN-γ) production and their surface receptor expression in response to LPS activation was investigated. PTX free cultures served as a control. Results revealed that PTX can down-regulate all the above-mentioned immunological parameters in a dose-dependent manner. These findings might have far reaching clinical implications.**

**Key words:** Adhesion molecules, Cytokines, Lymphokine activated killer cells, Natural killer cells, Pentoxifylline

### Introduction

Cytokines and adhesion molecules play an important role in normal immune regulation,[1,2] haematopoieses[3–5] and inflammatory responses.[6] Imbalances in the production of cytokines, particularly those affecting inflammation response, can have profound effects on immunoregulation and may contribute to pathogenesis of numerous diseases.[6,7] High physiological levels of cytokines and enhanced expression and production of soluble adhesion molecules have been implicated in a number of clinical situations.[6] Recently, considerable interest has also been focused on the adhesion molecules,[8] particularly for their role in pathogenesis during inflammatory processes. Cytokines are known to activate[4,9] a wide variety of killer lineage cells, induce cytokine production and adhesion molecules expression. PTX has been shown[10,11] to modulate neutrophil and monocyte functions. In the present study, to understand the mechanism and immuno-modulatory potential, the effects of PTX on the activation of peripheral blood mononuclear cells (PBMC), killer cells function, production of cytokines, surface receptor expression of cytokines and adhesion molecules was examined.

### Materials and methods

*Pentoxifylline:* Lot 61H0058 was purchased from Sigma (St Louis, MO, USA) and a stock solution of 1 mg/ml was prepared in sterile physiological saline (Abbott Laboratories, IL, USA). Further working dilutions were made in RPMI-1640 tissue culture medium (Gibco, Grand Island, NY, USA).

*Lipopolysaccharide:* Serotype 026.86 was purchased from Sigma, USA. Stock solution and working dilutions were prepared in RPMI-1640 medium just before use in various experiments.

*Cytokines:* Recombinant human IL-6 was purchased from Genzyme Corp. (Boston, MA, USA), recombinant human IL-2 (Batch No. C151597-02, R0330) was kindly provided as a gift from Hoffman–La Roche, Switzerland. All the cytokines were reconstituted in RPMI-1640 and were diluted further immediately before use.[4]

*Collection and preparation of peripheral blood mononuclear cells:* Samples (20–30 ml) of whole blood from healthy individuals of either sex were collected into heparinized tubes. PBMC were isolated by Ficoll–Hypaque fractionation, cell count and viability was carried out using the Trypan blue dye (0.02%) exclusion method. PBMC were finally suspended into RPMI-1640 medium supplemented with 25 mM Hepes and glutamine, streptomycin 100 μg/ml, fungizone 25 μg/ml, penicillin 100 U/ml and 10% AB human serum.

*Activation of monocytes and PBMC:* Monocytes were isolated as described previously.[12,13] Six replicate cultures of either φM (98% as judged by non-specific esterases staining) or PBMC were set

*A part of this work was presented at 8th International Congress of Immunology Meeting, Budapest, Hungary, 23–28 August 1992.

© 1993 Rapid Communications of Oxford Ltd up for 72 h in complete RPMI-1640 medium at 37°C, 5% $CO_2$ as follows:

(a) PBMC ($1 \times 10^6$/ml) + IL-2 100 U or IL-6 100 U

(b) $\phi$M ($1 \times 10^6$ ml) alone or + LPS 10 $\mu$g/ml

(c) PBMC alone or + LPS 10 $\mu$g/ml

(d) PBMC + LPS + PTX (10–250 ng/well/250 $\mu$l)

Effects of PTX on $\gamma$IL-2, IL-6 and LPS induced proliferation of PBMC was measured by [$^3$H]TdR uptake in culture (a) after 72 h. Culture (b) containing $\phi$M was used in antibody dependent cytotoxicity (ADCC) type killer assays. Fresh PBMC alone or after co-culturing with $\gamma$IL-2 1000 U/1 $\times 10^6$ PBMC/ml for 3 days were used as effector cells for NK and LAK cell assays respectively. PBMC from cultures (c and d) were used to examine surface receptor expression for various cytokines and adhesion molecules, whereas supernatant from these cultures was used to quantitate various cytokines. Unstimulated cultures of PBMC and $\phi$M alone or with PTX were used as a control.

*$\phi$M, NK and LAK cell mediated killer cell assay:* Using K562 cells (erythroleukaemia cells, NK sensitive target) or Daudi cells (Burkit lymphoma, NK resistant target) as described previously[14–16] in a $^{51}$Cr release killer assay ($\phi$M 18 h assay using K562 target cells), or (NK and LAK cells in a 4 h assay), specific cytotoxicity was measured at various effector:target ratios (100:1, 50:1, 25:1, 12.5:1) for $\phi$M, NK or LAK cells. Spontaneous and maximum $^{51}$Cr releases were also set up. Results were computed as follows:

$$\% \text{ specific cytotoxicity} = \frac{^{51}\text{Cr CPM exp} - {}^{51}\text{Cr CPM spontaneous}}{^{51}\text{Cr CPM max} - {}^{51}\text{Cr CPM spontaneous}} \times 100$$

*Cytokine quantitation:* Highly sensitive ELISA kits from Endogen (Boston, MA, USA), Genzyme Corp. and R & D System (USA) were used to quantify IL-1$\alpha$, IL-2, TNF-$\alpha$, IL-6 and IFN-$\gamma$ from the supernatant collected from PBMC culture under various conditions as described in the methodology. Manufacturer's guidelines were followed to measure these cytokines.[4,17] Supernatant from PBMC cultured without LPS and PTX were considered negative controls, whereas supernatant from PBMC plus LPS were considered positive controls.

*Cytokine surface receptor assay:* Using fluorokine kits (British Biotechnology, R & D System, UK) surface receptors for IL-1$\alpha$, IL-2 and TNF-$\alpha$ were measured on resting or activated (with LPS) PBMC with or without co-culturing with PTX as described earlier in the methodology. Manufacturer's guidelines were followed to measure the receptor of the cytokine using fluorescence microscopy (Zeiss, Germany) or FACS (Flow Cytometry). Results were expressed as a percentage of positive cells compared with unactivated PBMC.

*Expression of adhesions molecules LFA-1, Mac-1 and ICAM-1:* Adhesion molecules (LFA-1, Mac-1 and ICAM-1) on LPS activated PBMC (with or without PTX) were measured by indirect labelling. PBMC were first incubated with anti-LFA-1 (1:20 dilution), anti-Mac-1 (1:25 dilution) or anti-ICAM-1 (1:50 dilution, Bender & Co., Vienna, Austria) monoclonal antibody for 1 h at room temperature. After washing secondary labelling was carried out using goat anti-mouse FITC antibody (1:25 dilution) for 45 min. Negative controls were set up by excluding primary antibody. PBMC was analysed with FACScan (Becton and Dickinson, USA) and fluorescence microscopy (Zeiss, Germany). Results were expressed as percentage of positive cells.

*Statistical evaluation:* The results were expressed as the mean $\pm$ the standard error (S.E.). The difference between mean values was considered significant at $p \leq 0.05$. This was determined with Student's *t*-test when comparing two sets of means. A two-way analysis of variance was employed for comparing percentage of specific cytotoxicity under two different conditions, inclusive of all effector:target ratios.

### Results

*Effects of PTX on and cytokine induced activation of PBMC:* PBMC activation either with LPS or cytokines, that is IL-2 and IL-6, was down-regulated in a dose-dependent manner when PTX was added to the cultures at the initiation of the cultures (Fig. 1) indicating suppressive effect on the pre-effector level. Addition of PTX to unstimulated PBMC had no significant effect on [$^3$H]TdR incorporation. Once the PBMC had achieved their maximum activations, additions of PTX was somewhat less effective (results not shown). A maximum suppression was achieved at 250 ng/well/250 $\mu$l of PTX concentration ($p < 0.002$ for IL-2; $p < 0.023$ for IL-6 and $p < 0.005$ for LPS).

*Effects of PTX on the $\phi$M mediated cytotoxicity:* The activated macrophages mediated cytotoxicity was down-regulated in a dose-dependent manner (Fig. 2) only when PTX was added to the cultures at the initiation of cultures. Maximum suppression in $\phi$M cytotoxicity was only observed at 72 h, results of 24 h and 48 h cytotoxicity assays in the presence of PTX (data not shown) did not show significant suppression. Addition of PTX (100 ng/well/250 $\mu$l) to similar cultures after 48 h or

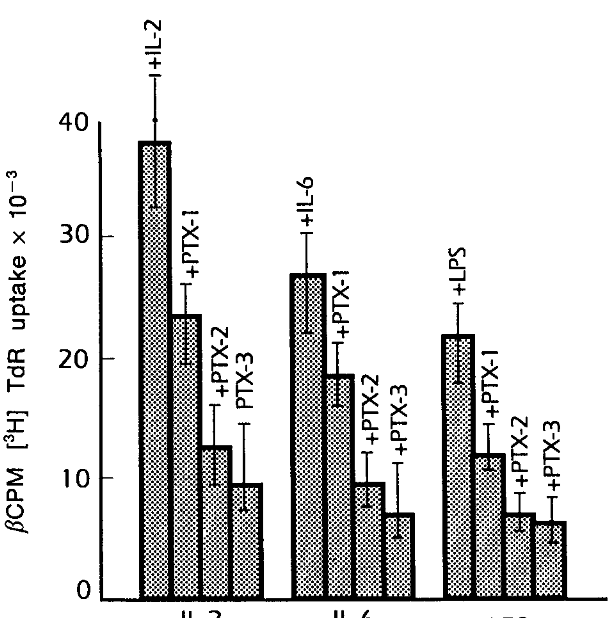

FIG. 1. Effects of PTX (PTX-1 = 50 ng, PTX-2 = 100 ng, PTX-3 = 250 ng) on IL-2, IL-6 and LPS induced proliferation of PBMC. PTX was introduced into the cultures at 0 h. Addition of PTX (250 ng) had no significant effect on the [$^3$H]TdR incorporation of unstimulated (control) PBMC (data not shown). The results are mean ± S.E. of three different experiments.

72 h was less effective, whereas a minor (5–7%) suppression was observed when PTX was added at 24 h to LPS stimulated cultures of $\phi$M. A high significant suppression ($p < 0.005$) at a 100:1 effector:target ratio was observed in the presence of PTX ≥ 100 ng/well when added at 0 h to the cultures.

*Effects of PTX on NK and LAK cell function:* Addition of PTX (10–250 ng/well/250 μl) to both NK and LAK cell assays at the initiation of cultures resulted in a dose-dependent suppression (Fig. 3). A highly significant suppression $p < 0.001$ was noted for both NK and LAK cells at 250 ng PTX/well or (1 μg/ml) concentration. Addition of similar dose of PTX during LAK cells assay, however, was not effective (result not shown).

*Effects of PTX on cytokine production and their receptor expression:* PBMC activated with LPS for 48 to 72 h with or without PTX when analysed for the surface receptor expression of IL-1α, IL-2, TNF-α and the cytokine production of IL-1α, IL-2, TNF-α, IL-6 and IFN-γ in the supernatant of these cultures indicated (Figs 4 and 5) a highly significant ($p < 0.001$) suppression of cytokine production and their surface receptor expression in the presence of ($p < 0.05$) PTX. Surface receptor expression and production of IL-6 was only noticed at the higher concentration of PTX (0.4 μg/ml).

*Effects of PTX on adhesion molecule expression:* Activated PBMC when analysed for the expression of LFA-1,

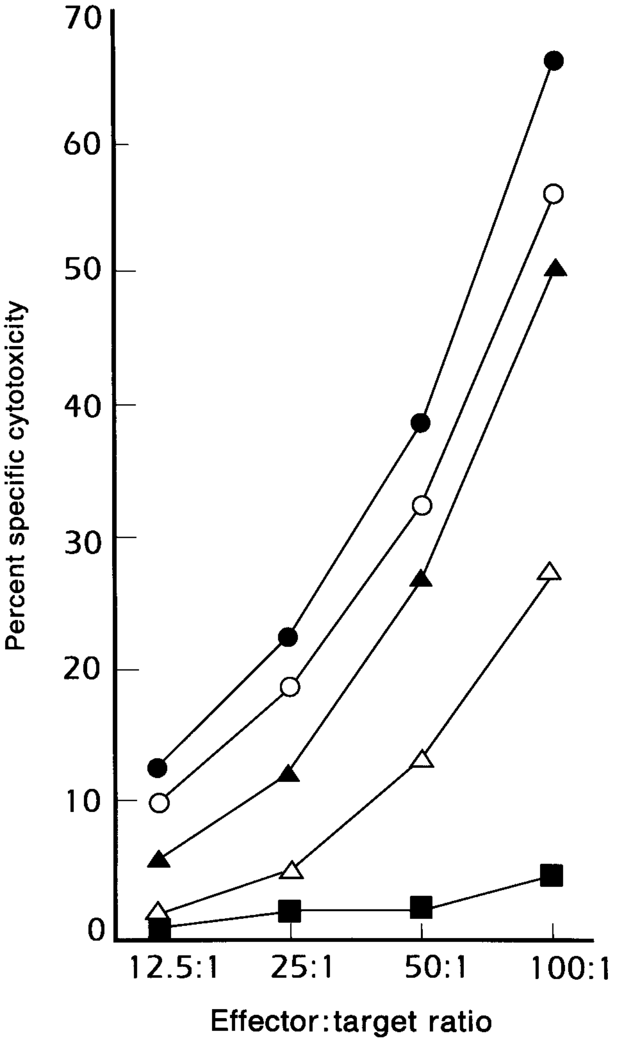

FIG. 2. Effects of various concentrations of PTX on $\phi$M mediated 18 h cytotoxicity assay (measured against K562 target). Each point represents the mean of triplicates with less than 5% variation. The results are representative of one experiment from a total of three. Addition of PTX to unstimulated $\phi$M had no significant effect (data not shown). ●, LPS activated $\phi$M; ○, +PTX 10 ng; ▲, +PTX 50 ng; △, + PTX 100 ng; ■, unactivated $\phi$M.

MAC-1, ICAM-1 revealed a dose-dependent suppression of LFA-1 and MAC-1 adhesion molecules whereas ICAM-1 expression was modulated only at higher concentrations of PTX (≥100 ng/$10^6$ PBMC/well) (Fig. 6).

### Discussion

Polymorphonuclear neutrophils (PMN) participate significantly during certain pathological states to induce a variety of cellular injuries.[18] Activation of PMN may lead to free oxygen radical production, enhance cytotoxicity via proteolytic enzymes and lead to increased adhesion to endothelial–cell lining.[19–21] Several cytokines are known to activate a wide variety of killer lineage cells, induce surface receptor expression, induce the production of adhesion molecules and lead to production of other

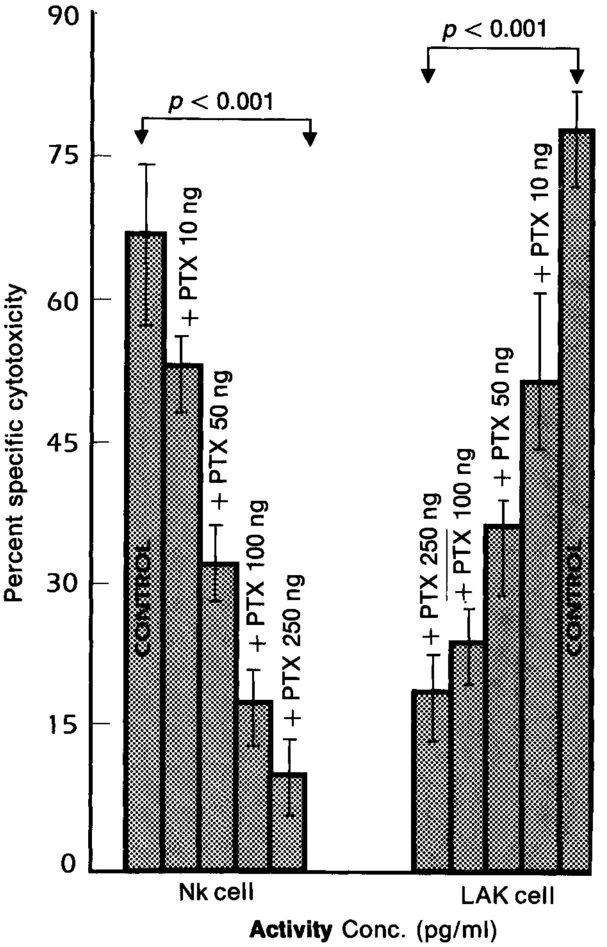

FIG. 3. Modulation of NK and LAK cell function by various concentrations of PTX. The results are the mean ±S.E. of three different experiments.

cytokines.[4,5] PTX mediated modulation of neutrophil functions has been reported in previous studies.[11,22,23] The authors[17] and others[24,25] recently showed that endotoxin induced cytokines i.e. IL-1α TNF-α and IL-6 are implicated in the pathophysiology of acute heat stroke patients. Imbalance in cytokine or their soluble receptor production, killer cell activation and adhesion molecule expression has also been implicated in a number of diseases.[26,27]

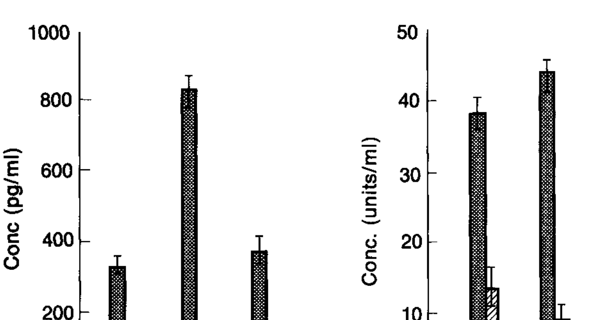

FIG. 4. Effects of PTX on *in vitro* cytokine production. The results are the mean ±S.E. of three different experiments. □, Control; ▩, LPS; ▨, PTX 100 ng/ml.

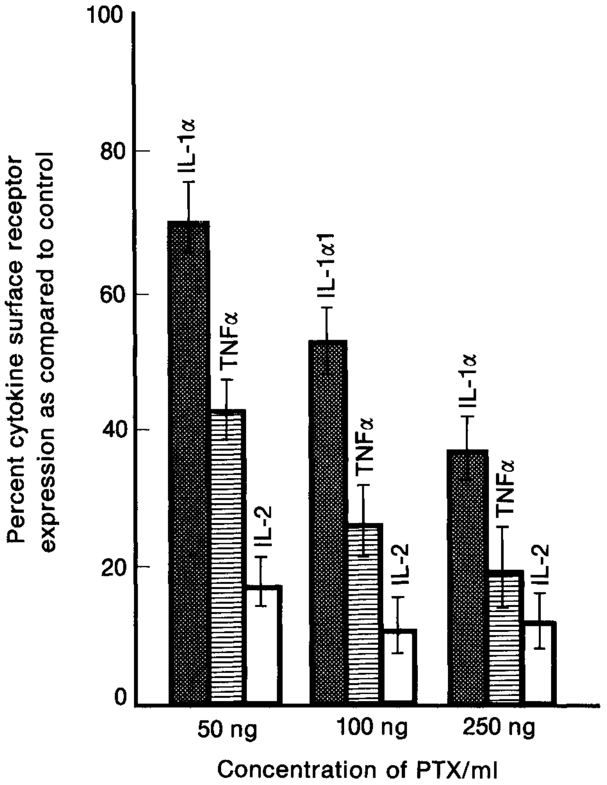

FIG. 5. Effects of PTX on the surface receptor expression on various cytokines. The results are the mean ±S.E. of three different experiments.

The aim of the present study was to analyse to what degree PTX interferes with various immune cell functions that are thought to play roles in a variety of normal and abnormal inflammatory states.

The results of this study clearly demonstrate that PTX can block activation and subsequent generation of killer lineage cells in a dose-dependent manner. LPS induced nonspecific activation of PBMC or cytokine mediated cytotoxicity, as measured either by using [$^3$H]TdR uptake or cytotoxicity assay, was significantly suppressed, when PTX was added at the initiation of PBMC

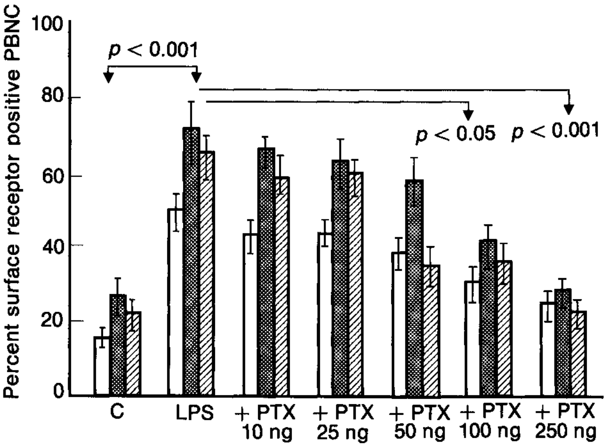

FIG. 6. Effects of PTX on the LPS induced surface adhesion molecules expression on PBMC on day 3. The results are the mean ±S.E. of three different experiments. □, LFA-1; ▩, Mac-1; ▨, ICAM1-1.

proliferation or killer cell assay. The significant suppression in mixed lymphocyte culture (MLR) and subsequent generation of allo-specific cytotoxic T-lymphocyte (CTL) killer cells (data not shown) was only achieved at a high concentration of PTX ($\geq 20$ $\mu$g/ml), when PTX was introduced into the culture at 0 h. Once the allo-specific CTL cytotoxic killer cells were generated, addition of PTX even at 10–20 $\mu$g/ml during the CTL assay was less effective. An early report[28] on a phase I—II human trial using PTX for the prevention of transplant related toxicity following bone marrow transplant (BMT), showed significant suppression in the level of circulating TNF-$\alpha$ and was associated with the reduction in morbidity and mortality in patients undergoing BMT. LPS induced cytokine (IL-1$\alpha$, IL-2, IL-6, TNF-$\alpha$ and IFN-$\gamma$) and surface receptor expression of IL-$\alpha$, IL-2 and TNF-$\alpha$ were significantly affected only when PTX was added at the beginning of the culture, indicating that once the maximum activation of PBMC had been achieved, PTX was no longer able to suppress the production of these cytokines. However, a recent study[29] showing a differential effect of PTX on TNF-$\alpha$ and IL-6 production *in vivo* in response to OKT3 monoclonal antibody in transplant patients clearly demonstrated a different mechanism of PTX action, depending on the cellular origin of these cytokines and the availability of accessory cells which can modulate the antigen presentation during induction of cytokine production. In the present study the effect of PTX on LPS stimulated PBMC, not on the pure macrophages population was measured. The amount of cytokines produced is the total amount secreted by T-lymphocytes and macrophages together, so a selective effect of PTX on either population of cells producing cytokines cannot be differentiated.

The expression of adhesion molecules, LFA-1, Mac-1 and ICAM-1, on PBMC was significantly affected by PTX at higher concentrations only. Modulation of various adhesion molecules on human endothelial cells activated with various cytokines was less affected using similar concentrations of PTX (R. S. Parhar and S. Al-Sedairy, manuscript in preparation) indicating that regulation of endothelial cell surface adhesion molecule expression could be different to that of PBMC. IL-1$\alpha$, IL-2, TNF-$\alpha$, IL-6 and IFN-$\gamma$ are implicated in the pathogenesis of several clinical settings including arthritis, septic shock, hyperthermia and organ transplantation. A high level of soluble ICAM-1 adhesion receptor has been reported in malignant melanoma patients.[30] The ability of PTX to suppress surface receptor expression of adhesion molecules merits further study to evaluate its role in malignant diseases. PTX has recently been shown to inhibit mRNA expression of TNF-$\alpha$[29] in PBMC and TNF-$\alpha$ and IL-1$\beta$ in HL-60 leukaemia cells.[31] Though the precise inhibitory mechanism of PTX at the molecular level is still elusive, its ability to down-regulate the cytokine surface receptor expression and cytokine production appears to be the key mechanism in down-regulating the activation of leukocytes macrophages, NK and LAK cell mediated cytotoxicity. Since the surface receptor expression of IL-1$\alpha$, IL-2, TNF-$\alpha$, IFN-$\gamma$ and adhesion molecules on killer lineage cells is related to cell activation and cytotoxic state, it is speculated that the mechanism of the PTX effect may be explained by its ability to down-regulate and suppress the mRNA of inflammatory cytokines which subsequently induce adhesion molecule expression and their production. The ability of PTX to down-regulate, modulate and suppress killer lineage cell function, cytokine and adhesion molecule expression and production, respectively, warrant studies in various clinical settings to evaluate its therapeutic benefits.

### References

1. Morstyn G, Burgess AW. Hemopoietic growth factors: a review. *Cancer Res* 1988; **48:** 5624–5632.
2. Metcalf D. The molecular control of cell division differentiation commitment and maturation in haemopoietic cells. *Nature* 1989; **339:** 27–30.
3. Broxmeyer HE, Li LU, Platzer E, Feit C, Juliano L, Rubin BV. Comparative analysis of influences of immune gamma, alpha and beta interferons on human multipotential (CFU–GEMM), erythroid (BFu-E) and granulyte macrophage (CFU–GM) progenitor cells. *J Immunol* 1983; **131:** 1300–1305.
4. Parhar RS, Ernst P, Sheth KV, Al-Sedairy ST. Anti-tumor cytotoxic potential and effect on human bone barrow GM–CFU of human LAK cells generated in response to various cytokines. *Eur Cytokine Netw* 1992; **3:** 299–306.
5. Patarroyo M. Leukocyte adhesion to cells: molecular basis, physiological relevance and abnormalities. A review. *Scand J Immunol* 1989; **30:** 129–164.
6. Dayer JM, Fenner H. The role of cytokines and their inhibition in arthritis. *Baillière's Clinical Rheumatology* 1992; **6:** 485–516.
7. Sewell KL, Trentham DE. Pathogenesis of rheumatoid arthritis. *Lancet* 1993; **341:** 283–286.
8. Springer TA, Anderson DC, Rosenthal AS, Rothelein R, *Leukocyte adhesion molecules: structure, function and regulation*. Springer-Verlag, 1990.
9. Parhar RS, Lala PK. Amelioration of B16F10 melanoma lung metastasis in mice by a combination therapy with indomethacin and interleukin-2. *J Exp Med* 1987; **165:** 14–28.
10. Sullivan GW, Caper HT, Novick JW, Mandell GL. Inhibition of the inflammatory action of IL-1 and TNF-$\alpha$ on neutrophil function by pentoxifylline. *Infection and Immunity* 1988; **56(7):** 1722–1729.
11. Hakim J, Mandell GL, Movick WJ. *Pentoxifylline and analogues: effects on leukocyte functions*. KARGER, Switzerland, 1990.
12. Parhar RS, Lala PK. Changes in the host natural killer cell population in mice during tumor development. 2. Mechanism of suppression of NK activity. *Cell Immunol* 1985; **93:** 265–279.
13. Nelson JAS, Parhar RS, Scodras JM, Lala PK. Characterization of macrophage subsets regulating murine natural killer cell activity. *J Leukocyte Biol* 1990; **48:** 382–393.
14. Parhar RS, Lala PK. Prostaglandin $E_2$-mediated inactivation of various killer lineage cells by tumor-bearing host macrophages. *J Leukocyte Biol* 1988; **44:** 474–484.
15. Lala PK, Parhar RS. Cure of B16F10 melanoma lung metastasis in mice by chronic indomethacin therapy combined with repeated round of IL-2: characteristics of killer cells generated *in situ*. *Cancer Res* 1988; **48:** 1072–1079.
16. Parhar RS, Kennedy TG, Lala PK. Suppression of lymphocyte alloreactivity by early gestational human decidua: characterization of suppressor cells and suppressor molecules. *Cellular Immunol* 1988; **116:** 392–410.
17. Bouchama A, Parhar RS, El-Yazigi A, Sheth K, Al-Sedairy ST. Endotoxemia and release of tumor necrosis factor and interleukin-1$\alpha$ in acute heat stroke. *Am J Appl Physiol* 1991; **70(6):** 2640–2644.
18. Movat HZ, Cybulsky MI, Colditz IG, Chan MKW, Dinarlleo CA. Acute inflammation in grain-negative infection: endotoxin interleukin-1, tumor necrosis factor and neutrophil. *Fed Pro* 1987; **46:** 97–104.
19. Klebanoff SJ, Vadas MA, Harlan JM, Sparks LH, Gamble JR, Agosti JM,

Waltersdropni AM. Stimulation of neutrophils by tumor necrosis factor. *J Immunol* 1986: **136**: 4221–4225.
20. Jason HE, Tahrog JD. Mechanism of disruption of the articular cartilage surface in inflammation: neutrophil elastic increase availability of collagen type 11 epitopes for binding with antibody on the surface of articular cartilage. *J Clin Invest* 1991; **87**: 1531–1536.
21. Smith CW, Marlin SD, Rothelein R, Toman C, Anderson DC. Co-operative interaction of LFA-1 and Mac-1 with intercellular adhesion molecules-1 in facilitating adherence and transendothelial migration of human neutrophil *in vitro*. *J Clin Invest* 1989; **83**: 2008–2012.
22. Mandel GL, Novick WJ. *Pentoxifylline and leukocyte function*. Somerville, New Jersey: Hoechst-Roussell Pharmaceuticals, Inc., 1988.
23. Josaki K, Cortrino J, Kristie J, Krause PL, Kreutzer DL. Pentoxifylline induced modulation of human leukocyte function *in vitro*. *Am J Path* 1990; **136**: 623–630.
24. Dinarello CA. The biology of interleukin 1, comparison to tumor necrosis factor and the effect of the combination of these cytokines. In: Bonavida B, Gifford GE, Kirchner H, Old LJ, eds. *International Conference on Tumor Necrosis Factor and Related Cytotoxins (Heidelberg, 14–18 September, 1987)*. Basel: Karger, 1988; 154–159.
25. Cannon JG, Tompkins RG, Gelfand JA *et al.* Circulating interleukin-1 and tumor necrosis factor in septic shock and experimental endotoxin fever. *J Infect Dis* 1990; **161**: 78–94.
26. Arend WP, Dayer JM. Cytokins and cytokine inhibitors or antagonist in rheumatoid arthritis. *Arthritis and Rheumatism* 1990; **33**: 305–315.
27. Aderka D, Englemann M, Hornick V. Increase serum level of soluble cancer patients. *Cancer Res* 1991; **51**: 5602–5607.
28. Bianco JA, Applebaum FR, Nemunaitis J *et al.* Phase I–II trial of pentoxifylline for the prevention of transplant-related toxicities following bone marrow transplantation. *Blood* 1991; **78**: 1205–1211.
29. Schandene L, Vanderbussche P, Crusiaux A *et al.* Differential effect of pentoxifylline on the production of tumor necrosis factor alpha (TNF-$\alpha$) and interleukin-6 (IL-6) by monocytes and T cells. *Immunology* 1992; **76**: 30–34.
30. Giavazzi R, Chirivi RGS, Garofalo A. Soluble intracellular adhesion molecule 1 is released by human melanoma cells and is associated with tumor growth in nude mice. *Cancer Res* 1992; **52**: 2628–2630.
31. Weinberg JB, Mason SN, Worthman TS. Inhibition of tumor necrosis factor-A (TNF-$\alpha$) and interleukin-1$\beta$ (IL-1$\beta$) messenger RNA (mRNA) expression in HL-60 leukemia cells by pentoxifylline and dexamethosome: disassociation of acivicin induced TNF-$\alpha$ and IL-1$\beta$ mRNA expression from acivicin-induced monocytoid differentiation. *Blood* 1992; **79**: 3337–3343.

ACKNOWLEDGEMENTS. The authors wish to thank Mrs Nazma Hannan and Shannaz Siddiqui for their assistance in some experiments and Mrs Jennifer M. Almazan for her secretarial help in preparing this manuscript. We also wish to thank Aaron A. A. Kwassi for his help in reviewing the manuscript.

**Received 22 June 1993; accepted in revised form 9 August 1993**
